# Effects of Transcranial Direct Current Stimulation Treatment for Anorexia Nervosa

**DOI:** 10.3389/fpsyt.2021.717255

**Published:** 2021-10-06

**Authors:** Silvie Baumann, Tadeáš Mareš, Jakub Albrecht, Martin Anders, Kristýna Vochosková, Martin Hill, Josef Bulant, Anna Yamamotová, Ota Štastný, Tomáš Novák, Petra Holanová, Alena Lambertová, Hana Papežová

**Affiliations:** ^1^Department of Psychotherapy, National Institute of Mental Health, Klecany, Czechia; ^2^First Faculty of Medicine, Charles University, Prague, Czechia; ^3^Department of Psychiatry, First Faculty of Medicine, Charles University in Prague and General University Hospital in Prague, Prague, Czechia; ^4^Third Faculty of Medicine, Charles University, Prague, Czechia; ^5^Department of Steroid Hormones and Proteohormones, Institute of Endocrinology, Prague, Czechia; ^6^Department of Pediatrics and Inherited Metabolic Disorders, First Faculty of Medicine, Charles University and General University Hospital in Prague, Prague, Czechia; ^7^Department of Physiology, Third Faculty of Medicine, Charles University, Prague, Czechia

**Keywords:** self-perception, anorexia nervosa, brain stimulation, tDCS, transcranial direct current stimulation, EDE-Q, Zung scale of depression

## Abstract

**Background:** Anorexia nervosa (AN) is a life-threatening illness with poor treatment outcomes. Although transcranial direct current stimulation (tDCS) is a promising non-invasive brain stimulation method, its effect in patients with AN remains unclear.

**Objective:** This study investigated changes in maladaptive eating behavior, body mass index (BMI), and depression after 10 sessions of anodal tDCS over the left dorsolateral prefrontal cortex (DLPFC).

**Methods:** In this double-blind, randomized controlled trial, 43 inpatients with AN were divided to receive either active (*n* = 22) or sham (*n* = 21) tDCS over the left DLPFC (anode F3/cathode Fp2, 2 mA for 30 min). All patients filled the Eating Disorder Examination Questionnaire (EDE-Q) and Zung Self-Rating Depression Scale (ZUNG), and their BMI was measured. These values were obtained repeatedly in four stages: (1) before tDCS treatment, (2) after tDCS treatment, (3) in the follow-up after 2 weeks, and (4) in the follow-up after 4 weeks.

**Results:** Primary outcomes (EDE-Q) based on the ANOVA results do not show any between-group differences either after the active part of the study or in the follow-up. Secondary analysis reveals a reduction in some items of EDE-Q. Compared with sham tDCS, active tDCS significantly improved self-evaluation based on body shape (*p* < 0.05) and significantly decreased the need of excessive control over calorie intake (*p* < 0.05) in the 4-week follow-up. However, the results do not survive multiple comparison correction. In both sham and active groups, the BMI values improved, albeit not significantly.

**Conclusion:** We did not observe a significant effect of tDCS over the left DLPFC on complex psychopathology and weight recovery in patients with AN. tDCS reduced the need to follow specific dietary rules and improved body image evaluation in patients with AN. Tests with a larger sample and different positions of electrodes are needed.

**Clinical Trial Registration:** www.ClinicalTrials.gov, identifier: NCT03273205.

## Introduction

Anorexia nervosa (AN) is a serious life-threatening illness, which is found throughout all countries and all socioeconomic layers. AN is estimated to occur in 0.3–1.0% females and 0.1–0.3% males (1, 2). It is associated with the highest mortality rate among all mental disorders (5.1 deaths per 1,000 person/years), and the suicide rate for AN is 1.3 per 1,000 person/years (3). AN is a severe eating disorder characterized by deliberate weight loss induced and maintained purposefully by the patient. This disorder is associated with specific psychopathology, in which the intense fear of weight gain persists as an intrusive thought. Food restriction, excessive physical activity, and self-induced vomiting or diarrhea are usually present, resulting in malnutrition with secondary endocrine and metabolic changes. A distortion of self-perceived body image is present in many patients suffering from this condition (4). Standard treatment consists of regimen therapy (restriction of exercise and regular food intake), as well as psychotherapy and psychopharmacological support (antidepressants, anxiolytics, and antipsychotics). Despite medical progress and therapeutic advances, the efficiency of current treatment is only around 40% (5, 6). Therefore, further treatment options should be investigated.

Neurostimulation is a biological approach in psychiatry that includes intentional modulation of basic neuronal activity through targeted delivery of a stimulus (by a magnetic field, by an electric current, or both) (7). Transcranial direct current stimulation (tDCS) is a modern, well-tolerated method, which can be easily applied by trained personnel. It is assumed to be a safe technique (8–10), and the adverse effects are overall mild. The advantages of this method are low purchase costs and great therapeutic potential. In contrast to repetitive transcranial magnetic stimulation (TMS), the current delivered by tDCS is not considered strong enough to evoke an action potential in neurons. tDCS is commonly referred to as both a “subthreshold” and “neuromodulatory” stimulation technique. tDCS acts to modulate the rate of naturally occurring firing of neurons within the stimulated tissue (11). The stimulation shifts cortical excitability to a state of excitation or inhibition (12), depending on the position of electrodes. Anodal tDCS is associated with excitation of the stimulated brain area by depolarizing neurons and increasing the propensity for neuronal firing, whereas cathodal tDCS is associated with hyperpolarization (13, 14). Hundreds of trials in many areas (e.g., schizophrenia, post-stroke aphasia, and tinnitus) are ongoing due to advantages and potential of tDCS. Level B recommendation (probable efficacy) has been proved for treating of craving, major depressive disorder (MDD), and fibromyalgia (15). tDCS treatment of AN has not been consolidated and varies.

Unlike some other mental and neurological disorders, the exact neurobiological correlates of AN have not been fully elucidated. It is assumed that there is a dysfunction in brain reward and emotional circuits, and impaired balance between interoceptive and reward processing. It is known that patients with AN have increased cognitive control and ability to suppress hunger (16–18). There are several targets for invasive neuromodulation (deep brain stimulation) in AN: the nucleus accumbens, which is active on mood and reward pathways; the subcallosal cingulate gyrus as part of mood and anxiety pathways; and the ventral capsule/ventral striatum or anterior limb of internal capsule, which is included in anxiety and emotion pathways (19). Techniques of non-invasive brain stimulation (NIBS; TMS and tDCS) mainly focus on dorsolateral prefrontal cortex (DLPFC). The insula, which modulates reward processing, decision making, interoception, and mentalization (19), was used as a target of neuromodulation in one study only, with the authors using H-coil deep TMS (20). In another study, the dorsomedial prefrontal cortex (DMPFC), which is involved in self-regulation, cognitive and impulse control, decision making, and inhibition, was modulated (21).

DLPFC is involved in cognitive control, executive functioning, working memory, craving, and also control and regulation of the valence of emotional experiences (22–24). Extreme caloric restriction in AN can be a manifestation of a maladaptive mechanism for coping with anxiety, mood disorders, and other negative emotions (16). In the evidence-based guidelines on the therapeutic use of tDCS, anodal tDCS over the left DLPFC (with right orbitofrontal cathode) is promoted in the treatment of major depressive episodes without drug resistance (15). Similarly, tDCS over DLPFC (anodal left and cathodal right DLPFC) improves cognitive control over negative emotions in borderline personality disorder (25). Consequently, stimulation of DLPFC, important in emotion regulation, could reduce the need for dietary behavior. On the other hand, AN is known for excessive cognitive control (16), and DLPFC is considered to be one of the main areas of the cognitive control system (26, 27). Even recovered patients with AN have elevated cognitive control over reward processing (18). Based on these findings, the inhibition of DLPFC has potential to reduce excessive cognitive control in AN.

For AN, Hecht suggested placing the anode over the left prefrontal cortex and the cathode, either on the right homotopic region for non-selective serotonin reuptake inhibitor (non-SSRI)-medicated anorexics or on a non-cephalic site for SSRI-medicated anorexics (28). Khedr et al. (29) applied 10 sessions of anodal stimulation over the left DLPFC (anode F3, cathode extracephalic—over the contralateral arm) in an open-label study to seven treatment-resistant patients with AN. Five of the patients improved, as shown in results from questionnaires on eating pathology and depressive symptoms directly after the stimulations. Three of them were shown to have maintained the improvement at their 1-month follow-up assessments. Recently, Costanzo et al. (30) compared tDCS and family-based therapy (FBT) in patients with AN. They placed the anode on the left DLPFC and the cathode on the right DLPFC in study of 11 participants (three sessions a week, for 6 weeks). The second group of 12 patients received FBT in an open-label study. Body mass index (BMI) significantly increased in the tDCS group compared with the FBT group. No group differences were reported regarding eating disorder symptoms. Strumila et al. (31) stimulated nine patients with AN for 10 days, twice a day with the same placement (anode F3/cathode F4). They noticed reduced eating disorder and depressive symptoms after 20 stimulations and in 1-month follow-up.

We aimed to explore the effect of anodal tDCS over the left DLPFC and with the cathode over the right orbitofrontal region in the first randomized, double-blind, sham-controlled trial of 43 patients with AN. The primary objective of the study was to observe its effect on the eating psychopathology evaluated by Eating Disorder Examination Questionnaire (EDE-Q). The secondary objective was to collect clinical outcomes from stage 1 to 4, including BMI, Zung Self-Rating Depression Scale (ZUNG), tolerability, and safety of tDCS. As the anodal tDCS can influence the emotional regulation (25), we hypothesized that in the group of patients with active tDCS, there will be a greater weight gain and an improvement in eating behavior (e.g., less restriction and reduction of vomiting). Second, because the same protocol is used to treat MDD (15), we expected the rate of depression to decrease more significantly in the stimulated group.

## Materials and Methods

### Participants

All enrolled participants received standard treatment, as they were hospitalized in the Center for Diagnosis and Treatment of Eating Disorders in the Psychiatric Clinic of the First Medical Faculty, Charles University, Prague. The patients followed an intensive, comprehensive in-patient program with individual and group psychotherapy. The refeeding program was individually driven and depended on each patient's current BMI value. They used medication, if needed, over the study period. All participants obtained the tDCS treatment on top of the standard care.

**Figure 1:**
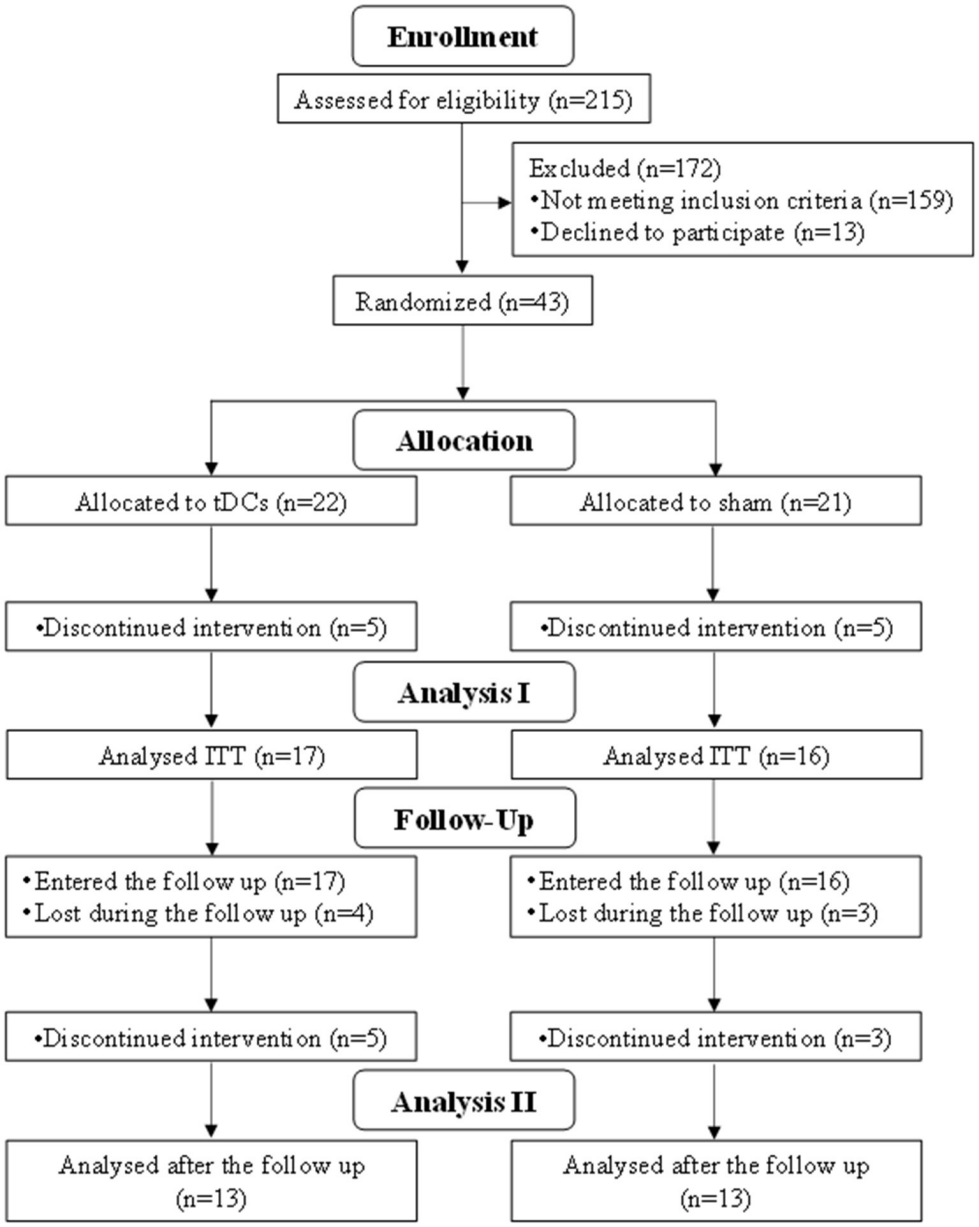
Enrollment to the study.

Inclusion criteria consisted of subjects between the ages of 18 and 65 with the diagnosis of AN according to the International Classification of Diseases 10th revision. Exclusion criteria included pregnancy or breastfeeding, a history of strong and frequent headaches, epileptic paroxysm and other severe neurological disorders, history of brain injury, and metallic objects within the neurocranium. Due to COVID-19 and certain technical and organizational challenges, we could not evaluate all consecutively hospitalized patients. All participants signed an informed consent and a General Data Protection Regulation processing agreement (approved by Ethical Committee No. 1955/16 S-IV). The recruitment period was from May 2017 to May 2020. Forty-three patients were selected for the study during this period. Thirty-nine patients were diagnosed with AN (90.7%), and four of them with atypical AN (9.3%). Eight of them were diagnosed with a personality disorder, seven with unipolar depression, and 10 with anxiety disorders, and five had a history of substance abuse (all of them sober for at least 3 months). The demographic data are shown in Table 1. The dataset had to be reduced due to a high dropout rate brought about by various reasons. Two patients (both from sham) kept breaking the rules of the Department (e.g., intentional vomiting and excessive exercising) and consequently were dismissed from the hospital, resulting in the termination in the study. Furthermore, two participants (one from sham and one from active) decided to leave the hospital against medical advice. Two females (sham group) requested to leave the study without disclosing the reason. Side effects represented the last reason (all four patients were from the active group): two patients left due to headache, another one was excluded following mood changes (toward hypomania), and one patient had troubles with blood sugar and an onset of diabetes (32). As a result, the data of 17 patients in the stimulated group and 16 in the sham (placebo) group remained eligible for the statistical analysis at the time of stage 2. Seven patients were lost to follow-up. One patient (active group) suffered from influenza during the third stage, and four participants (three from active and one from sham) finished the therapeutic program and left the Department before the termination of the study. Two patients (sham) withdrew from the study at stage 3. In the end, data of only 13 patients in each group were relevant for the statistical analysis (Figure 1).

**Table 1:** Baseline demographic and clinical characteristics of the study participants.

	**Active tDCS (*****n*** **=** **17)**		**Sham (*****n*** **=** **16)**		**Test**
**Characteristics**	**Median (quartiles)**	**Mean (SD)**	**Median (quartiles)**	**Mean (SD)**	**Statistics**
BMI	15.7 (14.7, 16.8)	16 (1.69)	17.3 (15.1, 18.3)	16.8 (2.47)	0.257*^a^*
EDE-Q total	96 (57, 131)	94.8 (40.2)	69 (33.3, 112)	75.9 (47.1)	0.183^a^
ZUNG	74 (70, 76)	71.6 (8.57)	72 (65.8, 79.5)	72.3 (11.4)	0.971^a^
Length of the illness (months)	48 (24, 84)	59.4 (46)	72 (46.5, 144)	98.6 (79.9)	0.176*^a^*
Number of psychiatric hospitalizations	2 (1, 3)	2.47 (2.1)	2 (1, 4)	4.19 (6.34)	0.583^a^
Number of psych. hospitalizations due to ED	1 (1, 3)	2.41 (2.12)	2 (1, 4)	4.06 (6.3)	0.480^a^
Age (years)	21 (20, 26)	23.7 (6.38)	26 (23.5, 33)	28.1 (7.95)	0.058^a^
**Characteristics**	***n*** **(%)**				
Depression	3 (17.6%)		4 (25%)		1.000*^b^*
Anxiety	3 (17.6%)		6 (37.5%)		0.259*^b^*
History of substance abuse	0 (0%)		5 (31.3%)		0.018*^b^*
Personality disorder	2 (11.8%)		3 (18.8%)		0.656*^b^*

*EDE-Q, Eating Disorder Examination Questionnaire; ZUNG, Zung Self-Rating Depression Scale; BMI, body mass index; ED, eating disorder*.

a *Mann–Whitney test*.

b *Fisher's exact test*.

### Study Protocol

The study protocol was approved by the Independent Ethics Committee on January 19, 2017, and is registered under number 1955/16 S-IV. The study was also registered in the ClinicalTrials.gov under the identifier NCT03273205. The design adhered to the latest version of the Declaration of Helsinki and International Council for Harmonisation (ICH)/Good Clinical Practice guidelines. It was a two-arm, double-blind, randomized controlled trial. We did not assess the blindness in our study. Participants were randomly assigned to active or sham groups by blocked randomization, and a block size of 4 was given.

We measured BMI, ZUNG, and EDE-Q in four stages: (1) before tDCS treatment, (2) after tDCS treatment, (3) in the follow-up after 2 weeks, and (4) in the follow-up after 4 weeks (Table 2). EDE-Q as a primary measurement for symptoms of AN consists of the four subscales demonstrating acceptable internal consistency with Cronbach's alphas ranging from 0.70 to 0.93 (33). Internal consistency (reliability) of ZUNG is reported in several studies with values around 0.8 (34, 35). The participants as well as the research team on-site remained unaware of the stimulation conditions until the last control.

**Table 2:** The timeline of the study.

**Stage**	**Timing**	**BMI**	**EDE-Q**	**ZUNG**
1	Before tDCS	X	X	X
2	After tDCS	X	X	X
3	2 weeks after	X	X	X
4	4 weeks after	X	X	X

The active protocol consisted of ten 30-min sessions of 2-mA anodal stimulation over the left DLPFC (F3 in 10–20 electroencephalography (EEG) system) with the cathode over the right orbitofrontal region (Fp2). The HDCstim portable programmable direct current stimulator made by Newronika s.r.l. (Milan, Italy) was used together with electrodes (anode 5 × 5 cm, cathode 6 × 8.5 cm) covered by hydratable holding bags soaked in saline (0.9%) to lower resistance. The current density was calculated at 0.571 A/m^2^. Modeling of electric fields was performed through SimNIBS software package (36) (Supplementary Figure 1).

For sham tDCS, the same protocol was used, but the device was automatically turned off after 30 s to mimic the typical initial sensation of tDCS and turned on for the last 30 s before the end of the session (so-called ramp-up and ramp-down, respectively). All other factors were the same for both groups (nutritional, pharmacological, and psychoeducational complex treatment “as usual”).

### Concomitant Treatment

The patients were enrolled in the study with their current pharmacotherapy. Due to the severity of their conditions, we did not build a washout period into our protocol. The medication is shown in Table 3.

**Table 3:** The concomitant treatment.

**Medication**	**Active tDCS (*n*)**	**Sham tDCS (*n*)**
Antidepressants	12	16
Antipsychotics	11	6
Benzodiazepines	4	3
Mood stabilizer (lamotrigine)	0	1
Pregabaline	0	1

### Statistical Analysis

The relationship between metric variables on the one side and the stage of the treatment and stimulation on the other side was assessed by ANOVA models. These models included the Subject factor explaining inter-individual variability, between-subject factor Stimulation (Stimulation vs. placebo), within-subject factor Stage (Stages 1, 2, 3, and 4), Stimulation × Stage interaction, and further factors such as comorbidities (MDD, anxiety, and personality disorders) and medication (antidepressants and antipsychotics) as covariates.

Before statistical testing, the parametric data were transformed utilizing power transformations toward normal distribution and homoscedasticity of data and residuals as described elsewhere (37, 38). The symmetry of the data distribution and the presence of outliers in the transformed data were evaluated using methods described in the literature (38–40). After analyses were performed, the obtained results were re-transformed by the recurrence formula to the original scale for their presentation.

Relationships between relevant variables in the first stage of the trial and their changes in the second stage of the trial on the one hand and the effect of stimulation on the other hand were evaluated by multivariate regression with a reduction of dimensionality known as orthogonal projection to latent structure (OPLS) (41–44). OPLS is capable of coping with the problem of severe multicollinearity in the matrix of explaining variables, while ordinary multiple regression fails to evaluate such data. In our OPLS models, the logarithm of the ratio of the probability that the subject underwent the stimulation to the probability that the subject was on placebo [logarithm of the likelihood ratio (LLR)] was chosen as a single dependent variable.

The variability in relevant explaining variables was separated into two groups of mutually independent components. The first one contained the variability of relevant explaining variables, which was shared with the effect of stimulation (the predictive component), while the orthogonal components explained the variability shared within the explaining variables.

The OPLS identified the relevant explaining variables and their combinations to estimate the effect of stimulation (Supplementary Figure 2). The relevant explaining variables were chosen using variable importance of projection (VIP) statistics. The statistical software SIMCA-P v.12.0 from Umetrics AB (Umeå, Sweden), which was used for OPLS analysis, detected multivariate non-homogeneities and tested the multivariate normal distribution and homoscedasticity (constant variance). The analysis was adjusted for multiple comparisons using Bonferroni's method. The respective algorithm is in Supplementary Figure 3.

## Results

Primary outcomes based on the ANOVA results do not show any differences between groups either after the active part of the study or in the follow-up. Secondary analysis (OPLS) reveals a reduction in certain items of EDE-Q in the 4-week follow-up. However, the results do not survive multiple comparison correction. In sham tDCS, several mood symptoms improved significantly (*p* < 0.01). In both sham and active groups, the BMI values improved, albeit not significantly.

Table 4 shows the OPLS model that analyzes the relationships between the effect of stimulation and monitored parameters at the beginning of the study as well as the differences between the values at the second and first stages of the study (Δ = *Stage 2* – *Stage 1*). There is a significant positive relationship between the stimulation and the changes in the overall score (*p* < 0.01) as well as in some individual questions of the ZUNG 5, 11, 12, and 20 (*p* < 0.01) and question 21 in EDE-Q (*p* < 0.05). This indicates that the sham group experienced a more pronounced decline in the aforementioned parameters. Table 4 also shows that more patients in the sham group took mirtazapine, smoked more cigarettes, and were older than patients in the active group. After Bonferroni's correction, only the following variables have *p* < 0.05: age, amount of cigarettes, single questions in ZUNG, and the total score in ZUNG.

**Table 4:** Relationships between the effect of stimulation (stimulated vs. non-stimulated patients, logarithm of the likelihood ratio, and LLR) and other parameters for the predictive component as evaluated by the OPLS model (for details, see *Statistical Analysis*).

	**OPLS model Predictive component**					**Ordinary multiple regression**		
	**Variable**	**Component loading**	**t-statistics**	**R** ***^*****a*****^***		**Regression coefficient**	**t-statistics**	
Relevant predictors (matrix **X**)	Trittico	−0.129	−1.68	−0.257		−0.071	−2.26	^*^
	Mirtazapine	−0.246	−2.65	−0.489	^*^	−0.134	−1.93	^*^
	Cigarettes	−0.178	−5.09	−0.354	^**^ ^†^	−0.091	−2.49	^*^
	Age	−0.256	−3.18	−0.508	^**^ ^†^	−0.105	−3.59	^**^
	EDE-Q, 4	0.161	1.53	0.320		0.095	1.82	
	EDE-Q, 15	0.248	1.65	0.492		0.116	1.83	
	ZUNG, 12	−0.227	−2.30	−0.451	^*^	−0.077	−2.69	^*^
	ΔEDE-Q, 21	0.123	2.33	0.245	^*^	0.078	2.26	^*^
	ΔEDE-Q, 28	0.255	1.72	0.506		0.094	3.36	^**^
	ΔZUNG, total	0.398	9.56	0.792	^**^ ^†^	0.148	5.94	^**^
	ΔZUNG, 5	0.287	3.76	0.571	^**^ ^†^	0.120	3.70	^**^
	ΔZUNG, 11	0.381	4.89	0.758	^**^ ^†^	0.136	3.76	^**^
	ΔZUNG, 12	0.355	5.78	0.706	^**^ ^†^	0.133	5.06	^**^
	ΔZUNG, 20	0.350	8.25	0.696	^**^ ^†^	0.149	5.91	^**^
(matrix **Y**)	Stimulation (LLR)	1.000	9.12	0.844	^**^			
**Explained variability**		71.2% (63.6% after cross-validation)						

*OPLS, orthogonal projection to latent structure; EDE-Q, Eating Disorder Examination Questionnaire; ZUNG, Zung Self-Rating Depression Scale*.

*EDE-Q, 4 = the need of excessive control over calorie intake; EDE-Q, 15 = number of episodes of overeating; EDE-Q, 21 = concerned when others see you eating; EDE-Q, 28 = uncomfortable feelings when others see your shape or figure (changing rooms, swimming, etc.); ZUNG, 5 = food intake as previously; ZUNG, 11 = clear mind; ZUNG, 12 = ability to do the things as before; ZUNG, 20 = hedonism*.

*aR = Component loadings expressed as correlation coefficients with predictive component*,

* *p < 0.05*,

** *p < 0.01*;

† *p < 0.05 after correction using Bonferroni's method;* Δ *symbolizes post-intervention change (Stage 2 – Stage 1)*.

*Sensitivity (95% CI) = 0.938 (0.717; 0.989); Specificity (95% CI) = 1.000 (0.772; 1.000). Cut-off probability = 0.5*.

Table 5 shows the OPLS model that analyzes the relationships between the effect of stimulation and parameters at the beginning of the study. In addition, it analyzes the differences between the situation at the final stage of the study and at the beginning of the study (Δ = *Stage 4* – *Stage 1*). Table 5 shows significant positive relationships between the changes in two questions in ZUNG (10, 16) and negative associations with two questions of EDE-Q (4, 23). Compared with sham tDCS, active tDCS significantly improved self-evaluation based on one's body shape (EDE-Q 23) and significantly decreased the need of excessive control over calorie intake (EDE-Q 4) in a follow-up after 4 weeks (*p* < 0.05). In sham tDCS, questions 10 (concerning fatigue) and 16 (ability to make decisions) improved significantly (*p* < 0.01). This shows that the active group experienced a more pronounced decline in the aforementioned EDE-Q changes but a less pronounced reduction in the ZUNG ones. In addition, more patients took mirtazapine and generally some antidepressants in the sham group. After Bonferroni's correction, the *p* < 0.05 holds true only for the question 10 in the ZUNG.

**Table 5:** Relationships between the effect of Stimulation (stimulated vs. non-stimulated patients, logarithm of the likelihood ratio, and LLR) and other parameters as evaluated by the OPLS model and ordinary multiple regression (for details, see *Statistical Analysis*).

	**OPLS model Predictive component**					**Ordinary multiple regression**		
	**Variable**	**Component loading**	**t-statistics**	**R** ***^**a**^***		**Regression coefficient**	**t-statistics**	
Relevant predictors (matrix **X**)	Mirtazapine	−0.266	−2.36	−0.588	^*^	−0.319	−4.99	^**^
	Antidepressants	−0.205	−2.00	−0.455	^*^	−0.176	−2.87	^*^
	EDE-Q, 1	0.179	2.04	0.395	^*^	0.103	1.49	
	EDE-Q, 2	0.288	4.64	0.638	^**^	0.147	1.26	
	EDE-Q, 3	0.212	4.26	0.469	^**^	0.012	0.19	
	EDE-Q, 4	0.300	4.78	0.664	^**^	0.130	1.61	
	EDE-Q, 5	0.172	4.98	0.380	^**^	−0.033	−0.54	
	EDE-Q, 8	0.165	2.09	0.365	^*^	0.023	0.30	
	EDE-Q, 10	0.195	3.24	0.431	^**^	0.012	0.25	
	EDE-Q, 11	0.174	2.27	0.384	^*^	0.008	0.18	
	EDE-Q, 12	0.154	3.51	0.341	^**^	−0.014	−0.30	
	EDE-Q, 18	0.202	2.72	0.448	^*^	0.040	0.40	
	EDE-Q, 20	0.142	1.97	0.314	^*^	−0.049	−0.78	
	EDE-Q, 22	0.234	2.88	0.517	^*^	0.124	1.80	
	EDE-Q, 23	0.234	3.89	0.517	^**^	0.103	1.68	
	EDE-Q, total	0.184	3.07	0.408	^**^	−0.068	−1.50	
	EDE-Q, restraint	0.264	4.99	0.584	^**^	0.066	1.66	
	EDE-Q, weight	0.149	2.50	0.330	^*^	−0.039	−1.54	
	ZUNG, 5	−0.207	−2.63	−0.459	^*^	−0.333	−3.54	^**^
	ΔEDE-Q, 4	−0.194	−2.73	−0.429	^*^	0.014	0.21	
	ΔEDE-Q, 23	−0.156	−2.00	−0.346	^*^	−0.024	−0.50	
	ΔZUNG, 10	0.160	3.78	0.354	^**^ ^†^	0.105	1.80	
	ΔZUNG, 16	0.266	2.11	0.589	^*^	0.198	2.29	^*^
(matrix **Y**)	Stimulation (LLR)	1.000	13.51	0.921	^**^			
**Explained variability**		84.7% (65.7% after cross-validation)						

*OPLS, orthogonal projection to latent structure; EDE-Q, Eating Disorder Examination Questionnaire; ZUNG, Zung Self-Rating Depression Scale*.

*EDE-Q, 1 = limitation of the amount of food intake; EDE-Q, 2 = periods without eating; EDE-Q, 3 = exclusion of favorite food; EDE-Q, 4 = the need of excessive control over calorie intake; EDE-Q, 5 = a desire to have an empty stomach; EDE-Q, 8 = overthinking about body shape and weight influences your concentration; EDE-Q, 10 = a fear of gaining weight; EDE-Q, 11 = feeling fat; EDE-Q, 12 = a desire to lose weight; EDE-Q, 18 = compulsive exercising; EDE-Q, 20 = feeling guilty for eating; EDE-Q, 22 = influence of the weight on self-estimation; EDE-Q, 23 = influence of the shape on self-estimation; ZUNG, 5 = food intake as previously; ZUNG, 10 = fatigue; ZUNG, 16 = ability to make decisions*.

*aR = Component loadings expressed as correlation coefficients with predictive component*,

* *p < 0.05*,

** *p < 0.01*;

† *p < 0.05 after correction using Bonferroni's method; Δ symbolizes post-intervention change (Stage 4 – Stage 1)*.

*Sensitivity (95% CI) = 1.000 (0.758; 1.000); Specificity (95% CI) = 1.000 (0.758; 1.000). Cut-off probability = 0.5*.

Table 6 shows the side effects of tDCS in our study. They are very similar to the side effects mentioned in literature (45, 46). The most common side effects were burning sensation under the electrodes and headache. Interestingly, one of the patients indicated an improvement of toothache; one mentioned remission of headache; and another patient noticed a decline in night sweating. On the contrary, there was an onset of type I diabetes mellitus in one patient with active tDCS (32).

**Table 6:** Summary of side effects.

**Total number of patients *n* = 43** **Sham *n* = 21** **Active tDCS *n* = 22**					
**Side effects**	**Sham (** ***n*** **)**	**Sham**	**Active tDCS (** ***n*** **)**	**Active tDCS**	**p-value Fisher's exact test**
Tingling	3	14.3%	3	13.6%	1.000
Itching	1	4.8%	3	13.6%	0.607
Burning sensation	3	14.3%	6	27.3%	0.457
Headache	4	19.0%	4	18.2%	1.000
Fatigue	2	9.5%	2	9.1%	1.000
Stitching	1	4.8%	1	4.5%	1.000
Pressure in the head	0	0.0%	1	4.5%	1.000
Acute mood changes	1	4.8%	2	9.1%	1.000
Pinching	3	14.3%	2	9.1%	0.664
Warm feelings under the electrodes	1	4.8%	0	0.0%	0.488
Metallic taste in the mouth	1	4.8%	0	0.0%	0.488
Phosphenes	2	9.5%	0	0.0%	0.233
Blurred vision	1	4.8%	1	4.5%	1.000
Scalp pain	0	0.0%	1	4.5%	1.000
Hyperglycemia with an onset of diabetes mellitus I	0	0.0%	1	4.5%	1.000
Dizziness	1	4.8%	0	0.0%	0.488
Burning in the eyes	0	0.0%	1	4.5%	1.000
Hand shaking	1	4.8%	0	0.0%	0.488
Neck stiffness	1	4.8%	0	0.0%	0.488
Tinnitus	1	4.8%	0	0.0%	0.488
Twitching of the eye	0	0.0%	1	4.5%	1.000
Remission of headache	1	4.8%	0	0.0%	0.488
Positive mood	2	9.5%	1	4.5%	0.607
Declined night sweating	1	4.8%	0	0.0%	0.488
Remission of toothache	1	4.8%	0	0.0%	0.488

## Discussion

In this study, we aimed to explore the effects of 10 sessions over the left DLPFC in patients with AN. The main analysis did not prove any significant effect on complex psychopathology and weight recovery in patients with AN. The secondary analysis indicates possible positive impact of tDCS treatment on questions 4 and 23 in EDE-Q. These findings indicate that active tDCS might reduce the urge to follow specific dietary rules and improves self-evaluation based on body shape. These factors are crucial for the long-term outcome of eating disorders.

Depression is often present in patients with AN as one of the comorbidities. According to the literature, tDCS is effective in the treatment of MDD (15, 47), which is why we expected some improvement of the active group in the ZUNG. However, just after the last stimulation (stage 2), the sham group had better results in the total score and in questions 5, 11, 12, and 20 in the ZUNG (*p* < 0.01). When we compared the first and last stages, there was a significant decrease in the sham group in questions 10 and 16 (*p* < 0.01 and *p* < 0.05). This may be explained by higher levels of MDD and higher doses of antidepressants, especially mirtazapine, in the sham group (Tables 1, 3, 4, 5). These could be important factors influencing our results. Another possible explanation is that in AN, affective difficulties are more likely to be secondary to primary eating pathology and increase with age (sham group is older). Thus, if the patients' core difficulties did not sufficiently change, neither did their moods.

The BMI values increased from the first stage to the last stage in both groups. It could be explained by regular food intake and strict control of the medical staff over the patients' eating habits. These might be confounding variables. For more accurate results, we would need a control group of inpatients receiving only the usual treatment.

The results of our study did not confirm promising studies that explored the effect of tDCS in AN. Two open-label trials were applied tDCS in seven and 10 patients (29, 31). Both of them showed an improvement in most of the patients. Costanzo et al. (30) tried to compare active tDCS and family psychotherapy and found that active tDCS was more effective. If we had not compared the active tDCS with the sham, our findings would have shown positive effects of active tDCS. However, in comparison with those of the sham group, most of our findings were not statistically significant.

The present study faces several limitations. First, the results could be influenced by different medications taken by the patients and higher antidepressant doses (mirtazapine in particular) taken by the sham group participants. The second limitation perhaps would be the small number of patients. We analyzed only 33 out of 43 patients enrolled in this trial, which is a borderline number for this kind of study. Third, the number of stimulations was rather small. Unfortunately, low compliance is typical for the diagnosis of AN, and the dropout rate equals to ~20–40% (48). In our study, the dropout rate was 23% up to stage 2, and 40% including the follow-up. To secure participation, we used only 10 stimulations, but it appears that the effects of tDCS can be cumulative (49, 50). Some studies demonstrate a long-term effect of tDCS in months or even years (51–54). We might not have reached the full potential of our protocol due to the small number of sessions in our study. Another important shortcoming of our study was a large number of variables (different ages, comorbidities, durations of the illness, and numbers of hospitalizations). Typically, the more chronic the illness, the lower the probability to recover.

To ensure appropriate application of tDCS, it is necessary to consider several factors, with the first being the target area, which should be selected based on neuroimaging studies and recent neuroscientific knowledge. Most of NIBS studies in AN targeted left DLPFC (19). Phillipou et al. published a systematic review of the neurobiology of AN and reported structural and functional brain imaging in AN. Nevertheless, the results are not definite due to many inconsistencies across study procedures, and the mechanism of this illness is still poorly understood (55). We can only presume that anodal modulation over the left DLPFC can bring some changes in patients with AN. There are several brain structures, which could be potentially suitable for the NIBS in AN. Phillipou et al. found distinctive eye movement abnormalities in patients with AN (56) and suggested neuromodulation of the inferior parietal lobe (57). As already mentioned at the beginning, also DMPFC (21) and insula (20) might be possible targets. The right DLPFC seems to be one of the fundamental regions for response inhibition (58, 59), which is one of the main cognitive processes. Based on the analyzed tDCS studies, it is mainly anodal tDCS over the right DLPFC, which improves the performance in healthy volunteers (60). As the patients with AN have an increased cognitive control, it would be worth trying cathodal tDCS over the right prefrontal cortex with anode extracephalic.

The electrode placement and their size are also important factors. The tDCS montage should be designed based on a current flow simulation executed beforehand. The reference electrode should be big enough, so that the current density under the electrode is insignificant, or another possibility is the use of several small return electrodes, which is even more efficient (61). That is why high-definition tDCS (four to eight electrodes), with more precise targeting, could be one of the possible future directions. One of the protocols for AN is already suggested by Phillipou et al. (57). Friehs et al. presented a simulation of current flow (performed with SimNIBS) when targeting the right DLPFC through two distinct setups. The first option encompassed a small anodal electrode (9 cm^2^) over the F4 position and cathode (9 cm^2^) extracephalic. The second option included 35 cm^2^ anode over F4 and 35 cm^2^ over the left supraorbital area. The presented difference is striking (60). Even if two studies target the same region, the different stimulation setups bring different effects. Also, our electrode placement may not have been optimal for maximum left DLPFC stimulation. The cathodal placement (Fp2) did not allow us to distinguish specific effect of left DLPFC excitation and decreased the stimulation focality (Supplementary Figure 1). It would have been more appropriate to place anodal electrode over the left DLPFC and cathodal electrode extracephalic as suggested by Hecht (28). An innovative placement was used by Frings et al. as they tried to influence cognition in healthy volunteers by a single session of tDCS. Instead of frequently used F3–F4 setup, a small electrode of 9 cm^2^ was placed over the left DLPFC and an electrode of 35 cm^2^ was placed over the parieto-occipital cortex. This alternative approach contrasting anodal vs. cathodal stimulation can help to distinguish inhibition vs. stimulation of the DLPFC (62).

Moreover, the current strength and current density should be taken into account. The smaller electrode, the lower the strength of the current necessary to achieve a constant value of the current density (61). For more practice guidelines in tDCS procedures, see Friehs et al. (60).

The psychiatric comorbidities are very common in people with eating disorders (>70%) (63). They usually share some similar characteristics; e.g., patients with AN have often obsessive compulsive disorder and MDD symptoms. Future tDCS studies in patients with AN could leverage more personalized protocols according to the predominant symptoms (19). A similar practice was used in electroconvulsive therapy (ECT) for treating AN [e.g., (64, 65)] or in deep brain stimulation studies (66). In the future, also individual placement guided by MRI (67) and a combination of tDCS and cognitive remediation or psychotherapy are another potential options for clinical research in AN. Although research in the identification of responders to repetitive TMS (rTMS) using EEG has yielded results (68), there may be underlying yet unidentified physiological factors that limit the response to tDCS. Research in this area also seems to be essential in order to personalize the treatment.

## Conclusion

Compared with sham, active treatment was not effective enough to cure complex psychopathology of AN. Our study suggests that tDCS may be beneficial for those with persisting body image disturbances or obsessive-compulsive calorie control, important factors for the remission achievement. More studies are necessary to confirm our results and specify clinical implementation additional to therapy as usual. Further research of efficacy of tDCS needs to concentrate on more specific and personalized indications.

## Data Availability Statement

The raw data supporting the conclusions of this article will be made available by the authors, without undue reservation.

## Ethics Statement

The studies involving human participants were reviewed and approved by Ethics Committee of the General University Hospital in Prague, No 1955/16 S-IV. The patients/participants provided their written informed consent to participate in this study.

## Author Contributions

SB, TM, OŠ, and KV: data acquisition. SB, TM, JA, MA, and HP: study design. TN, MH, JB, and SB: data analysis and interpretation. HP, PH, AL, and AY: project supervision. MH: contribution to the manuscript. SB: wrote the manuscript. TN, HP, TM, and JA: commented on the manuscript. All authors contributed to the article and approved the submitted version.

## Funding

This study was supported by Charles University Project GA UK No. 104121; MH CZ—DRO VFN64165; Q27/LF1; AZV 17-28905; and Progres Q35.

## Conflict of Interest

The authors declare that the research was conducted in the absence of any commercial or financial relationships that could be construed as a potential conflict of interest.

## Publisher's Note

All claims expressed in this article are solely those of the authors and do not necessarily represent those of their affiliated organizations, or those of the publisher, the editors and the reviewers. Any product that may be evaluated in this article, or claim that may be made by its manufacturer, is not guaranteed or endorsed by the publisher.
