# Supplementary material for: Effects of Transcranial Direct Current Stimulation Treatment for Anorexia Nervosa
Source: Front Psychiatry. 2021 Oct 6;12:717255. doi: 10.3389/fpsyt.2021.717255 (PMC8526853; doi:10.3389/fpsyt.2021.717255)
Supplement: Supplementary file 1 [file Data_Sheet_1.docx]

Supplementary Material


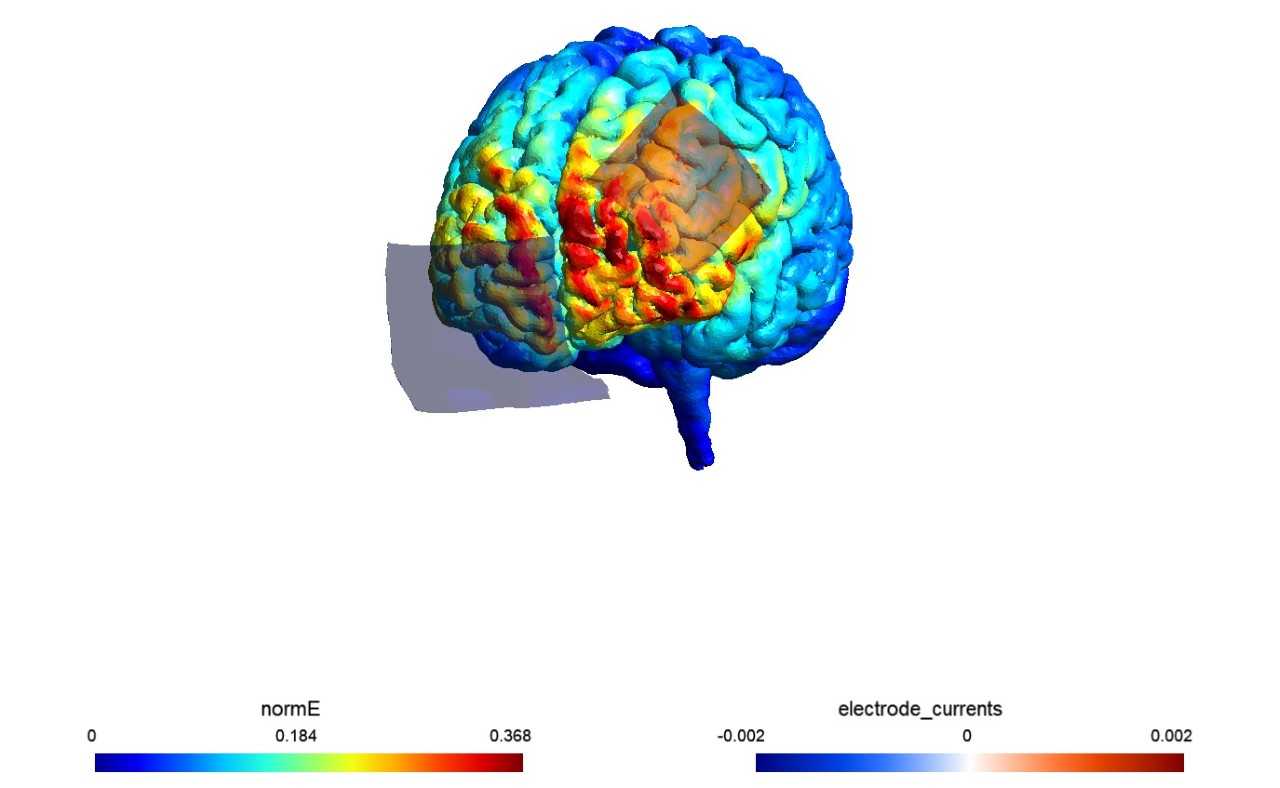


**Supplementary Figure 1**. A current flow simulation through SimNIBS

_
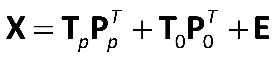
_ (2)

_
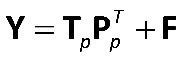
_ (3)

where **X** is the matrix with explaining variables and subjects, **Y** is the vector of dependent variable and subjects; **T**_p_ is the vector of component scores from the single predictive component and subjects extracted from **Y**; **T**_o_ is the vector of component scores from the single orthogonal component and subjects extracted from **X**; **P**_p_ is the vector of component loadings for the predictive component extracted from **Y**; **P**_o_ is the vector of component loadings for the orthogonal component extracted from **X** and independent variables; and **E** and **F** are the error terms.

**Supplementary Figure 2.** Definition of OPLS model

**Supplementary Figure 3**. The algorithm for analysis with use of OPLS model
